# Exploring the immunopotentiation of Chinese yam polysaccharide poly(lactic-co-glycolic acid) nanoparticles in an ovalbumin vaccine formulation *in vivo*

**DOI:** 10.1080/10717544.2017.1359861

**Published:** 2017-08-04

**Authors:** Li Luo, Tao Qin, Yifan Huang, Sisi Zheng, Ruonan Bo, Zhenguang Liu, Jie Xing, Yuanliang Hu, Jiaguo Liu, Deyun Wang

**Affiliations:** a College of Veterinary Medicine, Nanjing Agricultural University, Nanjing, PR China;; b Fujian Key Laboratory of Traditional Chinese Veterinary Medicine and Animal, Fujian Agriculture and Forestry University, Fuzhou, PR China

**Keywords:** Chinese yam polysaccharide, PLGA, nanoparticles, DCs, OVA, adjuvant

## Abstract

Biocompatible and biodegradable poly(lactic-co-glycolic acid) (PLGA) has been approved by the US Food and Drug Administration and has frequently been used to develop potential vaccine delivery systems. The immunoregulation and immunopotentiation of Chinese yam polysaccharide (CYP) have been widely demonstrated. In the current study, cell uptake mechanisms in dendritic cells (DCs) were monitored *in vitro* using confocal laser scanning microscopy, transmission electron microscopy, and flow cytometry. To study a CYP-PLGA nanoparticle-adjuvanted delivery system, CYP and ovalbumin (OVA) were encapsulated in PLGA nanoparticles (CYPPs) to act as a vaccine, and the formulation was tested in immunized mice. The CYPPs more easily underwent uptake by DCs *in vitro*, and CYPP/OVA could stimulate more effective antigen-specific immune responses than any of the single-component formulations *in vivo*. Mice immunized using CYPP/OVA exhibited more secretion of OVA-specific IgG antibodies, better proliferation, and higher cytokine secretion by splenocytes and significant activation of CD3^+^CD4^+^ and CD3^+^CD8^+^ T cells. Overall, the CYPP/OVA formulation produced a stronger humoral and cellular immune response and a mixed Th1/Th2 immune response with a greater Th1 bias in comparison with the other formulations. In conclusion, the data demonstrate that the CYPP-adjuvanted delivery system has the potential to strengthen immune responses and lay the foundation for novel adjuvant design.

## 1. Introduction

Vaccinations are applied to prevent infectious diseases caused by various viruses and bacteria and were first implemented on a wide scale more than 200 years ago with the introduction of the smallpox vaccine (Ulmer et al., 2006). Inadequate immunogenicity and safety concerns are key issues for consideration in designing vaccines, especially for those applied for prophylaxis. Subunit vaccines based on protein antigens are usually better tolerated and are regarded as safer alternatives to traditional vaccines, but are poorly immunogenic when used alone and therefore exogenous adjuvants are needed to enhance the resultant immune response (Broaders et al., 2009; Baumgartner & Malherbe, 2010; Sokolova et al., 2010).

Therefore, a crucial challenge for vaccine development is to design and create novel delivery systems that are safe and induce potent and long-lasting immune responses (Zinkernagel, 2003). An urgent problem involves determining the best way to present antigens more efficiently to APCs to subsequently induce their maturation and activities in conditioning the immune system for the successful development of adaptive immune responses (Mellman & Steinman, 2001).

In recent years, several delivery systems, including nanoparticles and microparticles, have been developed (Singh et al., 2007; Shi & Huang, 2009; Bachmann & Jennings, 2010). As one potential candidate, nanoparticulate-based adjuvants play a role in antigen delivery systems that facilitate the access of antigen to APCs and modulate the antigen presentation pathway, or as immune potentiators that enhance successful antigen-specific immune responses (Oyewumi et al., 2010; De Temmerman et al., 2011). DCs are considered the most efficient and specialized APCs with the capacity to stimulate strong immune responses (Pape et al., 2007).

PLGA is one of the most frequently used biocompatible and biodegradable polymers and is approved by the US Food and Drug Administration. PLGA can be formulated into nanospheres to encapsulate a wide range of bioactivators for sustained drug release in biological environments (Langer & Peppas, 1981). PLGA nanoparticles with a size range similar to microorganisms can easily undergo uptake by APCs (Peyre et al., 2004; Gomez et al., 2008). PLGA nanoparticles formulated to encapsulate a protein antigen result in very efficient and selective delivery to DCs in terms of co-encapsulated antigen and adjuvants (Reddy et al., 2007).

Chinese yam is a class of medicinal and edible plants, and Chinese yam polysaccharide (CYP), as the major functional component in Chinese yam, has a molecular weight of 16,619 Da and consists of glucose and galactose with a molar ratio of 1.52:1, and mainly contains 1, 3-linked-glc, 1-linked-gal, and 1, 6-linked-gal glycosidic bonds (Yang et al., 2015). In previous studies, CYP has been demonstrated to be efficacious for immune enhancement and demonstrates anti-tumor activity and immunomodulatory functions, and has been shown to decrease blood glucose levels (Hsu et al., 2003; Kim et al., 2003; Zhao et al., 2005). Nevertheless, CYP presents several obstacles to therapeutic utility including its short half-life. In order to capitalize on its immune enhancement properties and overcome its shortcomings, it was hypothesized that encapsulating CYP and antigen in PLGA nanoparticles would increase its antigen persistence and immune responses *in vitro* and *in vivo*.

The objectives of the current study were to investigate this novel type of adjuvant delivery system and assess the strength of the immune responses elicited by CYP-PLGA nanoparticle-based vaccine formulations that deliver OVA as a model antigen. Firstly, this study monitored the different cell uptake rates of DCs *in vitro* using CLSM, TEM and flow cytometry, which demonstrated that the CYPPs were most effective in undergoing cell uptake in DCs. Based on these *in vitro* results, the enhancement of antigen-specific immune responses by CYPPs encapsulating CYP and OVA protein (CYPP/OVA) was assessed, in comparison with free CYP mixed with OVA (CYP/OVA), blank PLGA nanoparticles encapsulating OVA (BP/OVA), OVA formulated with FIA (FIA/OVA), OVA alone, and a blank control (normal saline, NS). The immune response was evaluated by determining serum antibody titers, and the proliferation and cytokine expression of splenocytes following the administration of the various vaccine formulations to immunized mice. The results showed that CYPP/OVA stimulated stronger immune responses and a mixed Th1/Th2 immune response with a greater Th1 bias. The enhanced immune responses elicited by CYPP/OVA are directly attributable to the effective activation of DCs in the draining lymph nodes.

## 2. Materials and methods

### 2.1 Materials

PLGA (MW 15 kDa, monomer composition of lactide:glycolide of 75:25) was purchased from Jinan Daigang Biomaterial Co., Ltd. (Shandong, China). CYP (50% UV, No. CY150218) was purchased from Shanxi Ciyuan Biotechnology Co., Ltd. (Shanxi, China). Poloxamer 188 (Pluronic F68) was provided by Shanghai Yuanye Biotechnology Co., Ltd. (Shanghai, China). Recombinant mouse granulocyte macrophage colony-stimulating factor (rmGM-CSF) and recombinant mouse interleukin-4 (rmIL-4) were purchased from Peprotech Co. (USA). OVA, LPS, and PHA were supplied by Sigma-Aldrich (St. Louis, MO). FBS was purchased from Gibco Invitrogen (Carlsbad, CA). MTT was purchased from Amresco Co (Solon, OH). A Micro-BCA Protein Assay Kit was purchased from Pierce Biotechnology (Rockford, IL). A mouse cytokine ELISA kit was used to measure levels of IL-2, IL-4, IL-6, and IFN-γ and total IgG, IgG1, and IgG2a in mouse serum were measured using ELISA kits provided by Hangzhou MultiSciences Biotechnology Co., Ltd. (Hangzhou, China). An OVA-specific IgG ELISA kit for the assessment of mouse serum was obtained from R&D Systems Inc. (Minneapolis, MN). All fluorochrome-conjugated anti-mouse antibodies for flow cytometric use were purchased from eBioscience (San Diego, CA). All other reagents were of analytical grade.

### 2.2 Animals

BALB/c mice (6 weeks old, male and female) were purchased from the Comparative Medicine Center of Yangzhou University and acclimatized for 7 d before use. All mice were bred and housed in the Laboratory Animal Center of Nanjing Agricultural University, which maintained controlled conditions with a temperature of 25 ± 2 °C, a humidity of 60 ± 10%, and a 12:12-h light–dark cycle. Food and water were freely available to the mice. Each mouse was used once and treated according to the National Institutes of Health guidelines for the care and use of laboratory animals.

### 2.3 Preparation of empty and OVA-loaded NPs

The preparation of empty PLGA NPs and OVA-loaded NPs was based on the double emulsion solvent evaporation method (Luo et al., 2016). According to the response surface methodology, the optimal scheme was a volume ratio of the internal water phase to the organic phase of 1:9, a volume ratio of the primary emulsion to the external water phase of 1:10, and a concentration of F68 (w/v) of 0.7%. In brief, the water-in-oil primary emulsion was formed using a CYP solution in deionized water (20 mg/mL) as the internal water phase, which was added to the PLGA dispersed in acetone (20 mg/mL) as the organic phase. The mixture was sonicated using an ultrasonic cell disintegrator (XO92-IIN, Nanjing Xianou Biotechnology Co., Ltd., Nanjing, China) for 2 min (2 s on and 3 s off) at 130 W. The double emulsion (water-in-oil-in-water) was homogenized by pouring the primary emulsion into a Poloxamer 188 (F68) solution (0.7%, w/v) as the external water phase, followed by probe sonication for 2 min (2 s on and 3 s off) at 150 W. The residual organic solvent was removed using a rotary evaporator (Heidolph, Germany) for 30 min and the temperature was maintained at 55 °C, whereupon the nanoparticles were obtained. The BPs were prepared in the same way but the internal water phase did not contain CYP.

OVA-loaded NPs were produced using the same method but the internal water phase contained both OVA and CYP in deionized water. The BP/OVA was prepared in the same way but the internal water phase contained OVA but did not contain CYP.

### 2.4 Determination of OVA-EE and characterization of OVA-NP formulations

The OVA-EE in the OVA-NP formulations was measured using the Micro-BCA Protein Assay Kit (Pierce Biotechnology, Rockford, IL) in deionized water at 37 °C. In brief, to quantify the loaded OVA, the OVA-NP formulations were dissolved in 0.1 M NaOH and 0.1% SDS, incubated overnight at room temperature, and assessed using a Micro-BCA Protein Assay Kit, according to the protocol provided by the manufacturer. OVA dissolved in 0.1 M NaOH and 0.1% SDS was used to establish a standard curve, and empty BPs were used as a control. The OVA-EE was calculated by dividing the measured amount of encapsulated OVA by the theoretical amount assuming all was encapsulated. Data are expressed as the mean value of three independent experiments with the reported standard deviation.

The particle size (hydrodynamic diameter, in nm), size distribution (PDI) as a measure of the physical stability of the NPs, and the zeta potential (surface charge, in mV) of the CYPP, BP, CYPP/OVA, and BP/OVA were determined using a laser particle size analyzer (Hydro2000Mu, MAL1009117, Malvern Instruments, Malvern, UK). Deionized water was used as the dispersion medium, and the CYPP, BP, CYPP/OVA, and BP/OVA suspensions were assessed under almost the same humidity and temperature (25 °C). Each sample measurement was performed in triplicate simultaneously.

### 2.5 BMDC culture

BMDCs were harvested from the bone marrow of the femurs and tibias of BALB/c mice (6 weeks, male) as described in a previous study (Huang et al., 2013). Briefly, the mice were sacrificed and the surrounding muscle tissues of the bone were aseptically isolated. The complete bones were washed twice using PBS and soaked in alcohol for 3–5 min to completely remove the surrounding muscle tissues. BMDCs were cultured with 10 ng/mL of rmGM-CSF and 5 ng/mL of rmIL-4 (Peprotech) in complete media (RPMI 1640 media containing 100 IU/mL benzylpenicillin, 100 IU/mL streptomycin, and 12% heat-inactivated FBS) at 37 °C. The medium was changed every 44–48 h and incubated for 7 d.

### 2.6 Imaging the internalization of different formulations by DCs and the ultrastructure of DCs

To investigate DC uptake of the different drug formulations (including free CYP, BPs, and CYPPs), the BMDCs were harvested as noted above and added into 6-well plates with round coverslips. A total of 200 μL of the freshly prepared samples (mixed with FITC-OVA and incubated in the dark at 4 °C overnight) was incubated with the 7-d-old BMDCs for 12 h at 37 °C and 5% CO_2_. After incubation, the medium was immediately removed and cells were washed three times using PBS (pH 7.4). Freshly prepared 4% (w/v) paraformaldehyde was added into each well, and the cells were fixed for 20 min. Then, the cells were rinsed twice using PBS buffer. To label nuclei, the cells were incubated with the fluorescent dye DAPI (Sigma-Aldrich, St. Louis, MO) for 15 min. The cell samples were mounted with coverslips and sealed using 50% glycerin (Ding & Schwendeman, 2008). The DC uptake was visualized using CLSM (LSM 710, Zeiss, Oberkochen, Germany). The sample images were analyzed using ZEN lite 2014 confocal software on a per-pixel basis.

To verify whether the CYPPs were internalized by DCs, the DCs were cultured in 6-well plates for 7 d and were then cultivated for a further 48 h after treatment with the CYPPs. The cell morphology of the CYPP-treated DCs was observed using TEM (Model Tecnai 12, Philips Co., Ltd., Holland). For the TEM ultrastructural observations, CYPP-treated DCs were collected and fixed for 4 h at 4 °C in 2.5% glutaraldehyde, washed three times in 0.1 mol/L PBS, post-fixed, dehydrated, embedded, and cut into ultrathin sections (75 nm), and then the sections were finally viewed and images were recorded.

### 2.7 Immunophenotyping of the BMDCs

The BMDCs were obtained in the same way as in Section 2.5, and 1 × 10^6^ mL^−1^ cells were cultured in 6-well plates (Thermo Fisher Scientific, Inc., Waltham, MA) at 37 °C in a humidified 5% CO_2_ incubator. A blank control group and a LPS group as a positive control group (LPS group; the BMDCs were challenged using 5 µg/mL LPS) were designed for comparison. The DCs were cultured in an incubator under suitable growth conditions for 7 d and the medium was changed every 2 d. Then, the CYPPs were added to the DCs and incubated for 44 h. The DCs were collected and washed twice, then stained using anti-CD11c-APC, anti-MHC-II-PE-Cy5.5, anti-CD80-FITC, and anti-CD86-PE (BD Biosciences) in the dark at 4 °C for 30 min, which was in accordance with the protocol provided by the manufacturer. The cell pellets were dissociated by gentle pipetting for analysis using flow cytometry (BD FACSCalibur, Biosciences, Bedford, MA).

### 2.8 Immunization schemes and sample collection

The mice were randomly separated into six groups, housed individually, and immunized using CYPP/OVA (50 μg OVA +200 μg CYP in 0.2 mL of CYPP/OVA), CYP/OVA (50 μg OVA +200 μg CYP in 0.2 mL of CYP/OVA), and 50 μg of OVA in 0.2 mL of BP/OVA. The mice in the control groups were immunized using 50 μg of OVA in 0.2 mL of deionized water, or 50 μg of FIA/OVA and the mice in the blank control group were immunized using 0.2 mL of saline. The mice were subcutaneously injected with 0.1 mL in the neck and tail, at a dose of 0.2 mL in total, and they were inoculated two times at 1-week intervals.

Mice were randomly selected and sacrificed on days 14, 21, 28, 35, and 42 after the first immunization, at which time blood was collected using the ball-picking method, and splenocytes were harvested aseptically. Serum samples were isolated and stored at −70 °C.

### 2.9 Determination of mouse serum antibodies

Mouse serum total IgG antibody, OVA-specific IgG antibody, IgG1, and IgG2a were quantitatively measured using ELISA kits according to the instructions provided by the manufacturer. The serum was inactivated at 56 °C for 30 min before the assessment of the levels of OVA-specific IgG antibodies.

### 2.10 Splenic lymphocyte proliferation assay

A splenic lymphocyte proliferation assay was applied to evaluate OVA-specific lymphocyte activation, as demonstrated previously (Yuan et al., 2010).

Splenocytes (2 × 10^6^ cells/mL), activated with OVA (50 μg/mL) or not, or cultured with LPS or PHA, were seeded in quadruplicate (100 μL/well) in a 96-well plate and incubated at 37 °C in a humid atmosphere with 5% CO_2_ for 72 h. Cell proliferation was determined using an MTT assay. Briefly, 30 μL of MTT solution (5 mg/mL) was added to each well and incubation proceeded for 5 h. After centrifugation of the plates (3800 rpm, 10 min), untransformed MTT was removed carefully. Then, 100 μL of DMSO (Amresco) was added into each well and the plates were shaken for 6 min to completely dissolve the crystals. The absorbance was measured using a microliter enzyme-linked immunosorbent assay reader (Thermo Scientific Multiskan FC) at a wavelength of 570 nm (A_570_ value). The results are expressed as the proliferation index, based on the following formula:

$$Proliferation\;index\;(\%)=\frac{At-Ac}{Ac}$$

In this formula, *At* indicates the mean A_570_ value of the test group and *Ac* represents that of the control group.

### 2.11 Flow cytometric analysis of lymphocyte immunophenotype

At 28 d after the first immunization, splenic lymphocytes were harvested from the immunized mice, were incubated in 24-well plates (1 mL per well, 1 × 10^6^ cells/mL), and were stimulated using OVA (50 mg/mL) for 72 h. Then, the cells were stained using fluorescein-labeled antibodies, including anti-CD3e-FITC, anti-CD8-PE, and anti-CD4-APC antibodies (eBioscience). The stained cells were assessed for the percentages of CD3^+^CD4^+^ and CD3^+^CD8^+^ T cells using fluorescence-activated cell sorting (FACS).

### 2.12 Determination of cytokine levels

The splenic lymphocytes were harvested (as in Section 2.11) and cultured for 48 h. Meanwhile, the culture supernatants from the *in vitro* lymphocyte culture systems were collected through centrifugation. The concentrations of mouse IL-2, IFN-γ, IL-4, and IL-6 in the supernatants were measured according to the instructions provided with the quantitative ELISA kit (MultiSciences Biotech Co., Ltd.).

### 2.13 Analysis of DC activation in draining lymph nodes

DC activation in the mandibular lymph node, inguinal lymph nodes and popliteal lymph nodes was determined at 24 h and 48 h after vaccination using the various vaccine formulations, as described in Section 2.8. The lymph nodes were harvested and prepared as single cell suspensions. The cells were then washed, blocked, and stained using anti-CD11c-PE-Cy5.5, anti-MHC II-APC, anti-CD80-FITC, and anti-CD86-PE (eBioscience). Finally, flow cytometry was performed on a BD FACSCalibur flow cytometer.

### 2.14 Histomorphological observation of the spleen

At 35 d following the first immunization, the mice were sacrificed and the spleens of all groups were removed and then fixed overnight in 4% paraformaldehyde solution. After dehydration in a series of graded ethanol solutions, the samples were embedded in paraffin for subsequent sectioning using a microtome (Bo et al., 2016). Hematoxylin- and eosin-stained histological slides were subsequently visualized using an optical microscope (Nikon U-III Multipoint Sensor System).

### 2.15 Statistical analysis

Data are displayed as mean ± standard error (SEM) for each group. Duncan and LSD’s multiple range tests were used to determine the difference among groups. The FACS data analysis was conducted using FlowJo version 7.6.1 software. All data analysis was performed using the IBM SPSS Statistics 17.0 program (SPSS Inc., Chicago, IL). Differences in mean values were considered statistically significant at *p* < .05.

## 3. Results

### 3.1 Characterization of OVA-encapsulated NPs

**Figure 1.**
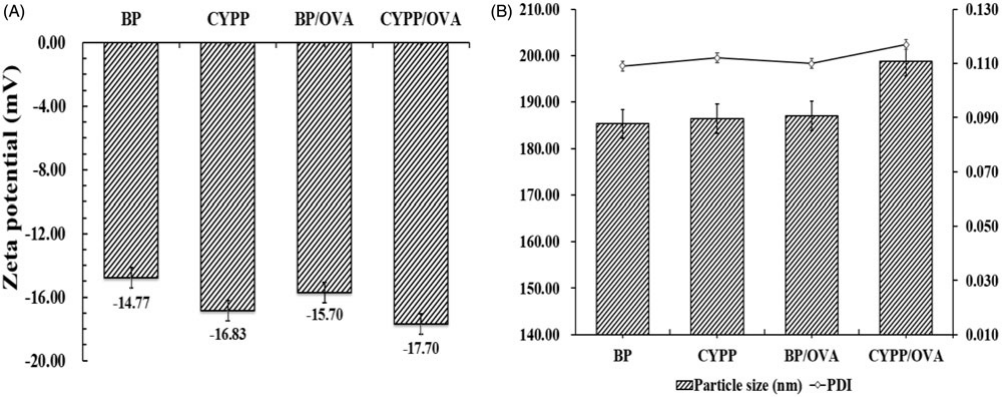
Stability of the NPs/OVA. Zeta potential of the NPs/OVA (A). Particle size and polydispersity index (PDI) of the NPs/OVA (B). Data are shown as mean ± SEM.

Figure 1(A,B) shows the respective zeta potential values, hydrodynamic particle size, and PDI of NPs/OVA in aqueous solution. The size of the CYPP/OVA (208.80 ± 0.71 nm) is bigger than that of the BP/OVA (186.43 ± 0.62 nm). Determination of the zeta potential demonstrated a negative surface charge for all the formulations, and the absolute value of the zeta potential of the CYPP/OVA (−17.79 ± 0.76) is higher than that of the BP/OVA (−14.77 ± 0.22). The zeta potential values of the CYPP/OVA and BP/OVA were treated as functional surface charge. In the current study, the unimodal size distribution with a mean particle size around 200 nm represented in terms of volume diameter, and the low mean PDI (<0.3) of BP/OVA and CYPP/OVA made a significant contribution to the colloidal stability of the NPs/OVA. The encapsulation efficiency of BP/OVA and CYPP/OVA was similarly high, at approximately 90.77% and 91.10%, respectively. According to these data, it is obvious that NPs/OVA were able to maintain colloidal stability and a high OVA-EE, which ensured the stability and effectiveness of the NPs/OVA.

### 3.2 Confocal morphology and ultrastructure of the DCs

**Figure 2.**
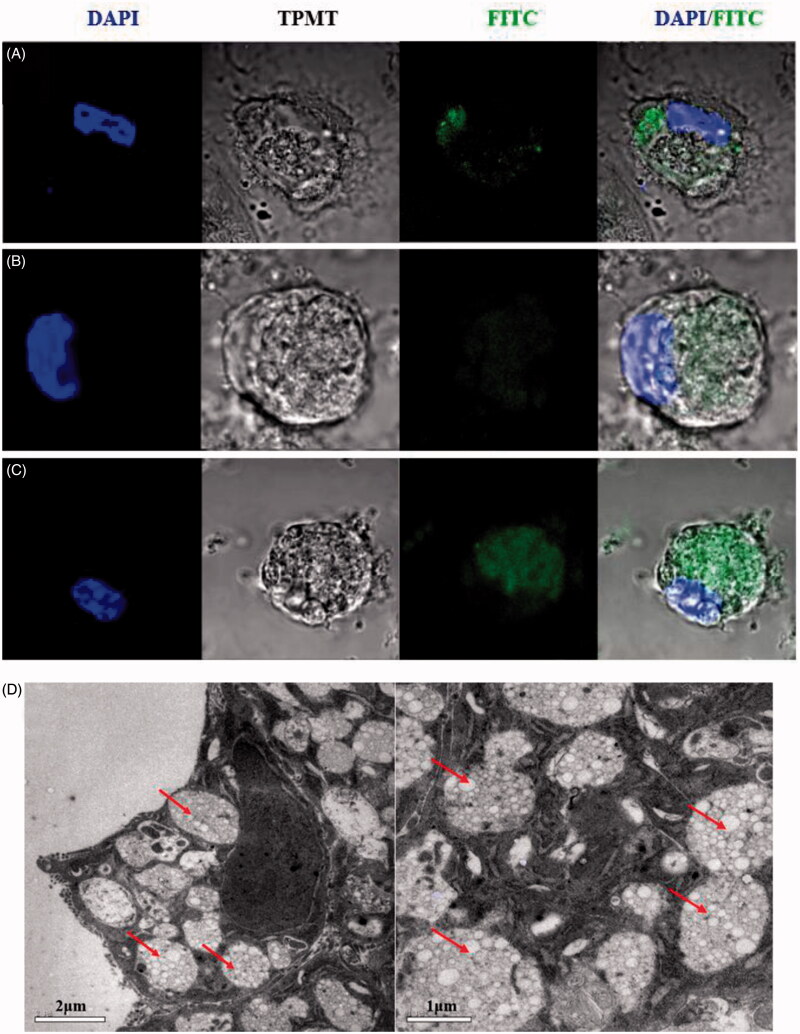
CLSM and TEM images of NPs and the cellular uptake of NPs. A total of 200 μL of FITC-OVA-stained NPs or CYP was incubated with 4 × 10^5^ DCs for 12 h. Confocal micrographs of DCs, in which different formulations were labeled using FITC-OVA (green color) and the nuclei (blue color), are stained using DAPI. The merged image shows the endocytosis of the freshly made formulations by the DCs. (A) CYP was labeled using FITC-OVA and co-cultured with DCs, (B) BPs were labeled using FITC-OVA and co-cultured with DCs, (C) CYPPs were labeled using FITC-OVA and co-cultured with DCs. Scale bar represents 5 μm. TEM micrographs of the CYPPs internalized by DCs and the arrow indicates the CYPPs (D). Scale bar represents 2 μm and 1 μm.

The efficacy of nanotherapies that depends on DCs is dependent on particle internalization (Look et al., 2014). To explore whether the three formulations were internalized by DCs *in vitro*, cellular uptake was measured using CLSM and TEM. As shown in Figure 2(A–C), the confocal microscopy pictures demonstrate that more abundant quantities of FITC-OVA-labeled CYPPs were taken up by the DCs compared with the BP NPs and free CYP. These results indicate that CYPPs are more easily internalized by DCs. As shown in Figure 2(D), the ultrastructure of the DCs, as assessed using TEM, shows that the NPs were internalized by the cells. The results of both the CLSM and TEM showed that PLGA nanoparticles were internalized and not merely adherent to the cell surface.

### 3.3 Evaluation of the effect of CYPPs on BMDC immunophenotype

**Figure 3.**
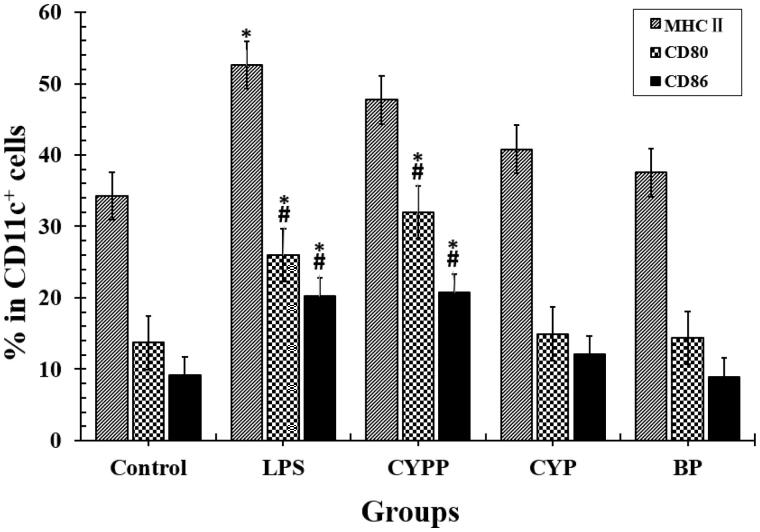
Investigation of the dendritic cell (DC) uptake of the different formulations, as assessed using flow cytometry. The percentage of MHC II^+^, CD80^+^, or CD86^+^ in CD11c^+^ DCs that had internalized the formulations. The results are representative of three separate experiments. *p < .05 vs. the BP group, while #*p* < .05 vs. the CYP group.

Previous experiments have demonstrated the viability of DCs exposed to CYPPs. The results show that the viability of the DCs upon *in vitro* stimulation, as detected using MTT assay, was more than 90% when the concentration of the vaccine formulation administered to the immunized mice was 500 μg/mL (250 μg/mL is used in the current article), which demonstrates no dose-dependent toxicity. BMDCs were incubated with equal concentrations of the formulations, to assess differential drug uptake and cell maturation. To perform this comparison quantitatively, flow cytometry was applied to evaluate the immunophenotype of the BMDCs. As shown in Figure 3, the surface marker expression was compared, and treatment using the CYPPs created greater improvements in the percentage of CD11c^+^ BMDCs that were positive for MHC II, CD80, and CD86, compared with cells exposed to CYP/OVA and BP/OVA.

### 3.4 Systemic immune responses in vaccinated mice

**Figure 4.**
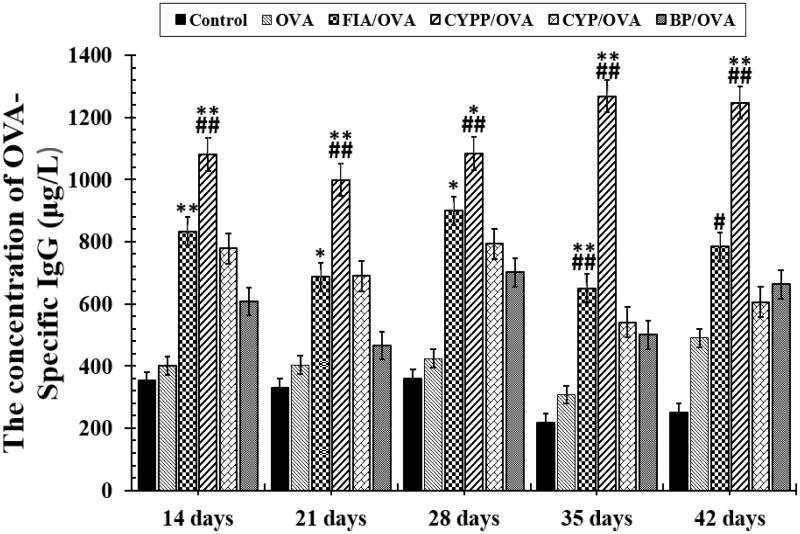
OVA-specific IgG antibody responses in mice immunized using different vaccine formulations. Data are expressed as the mean ± SEM. *p < .05 and **p < .01 vs. the BP/OVA group, while #*p* < .05 and ##*p* < .01 vs. the CYP/OVA group.

**Figure 5.**
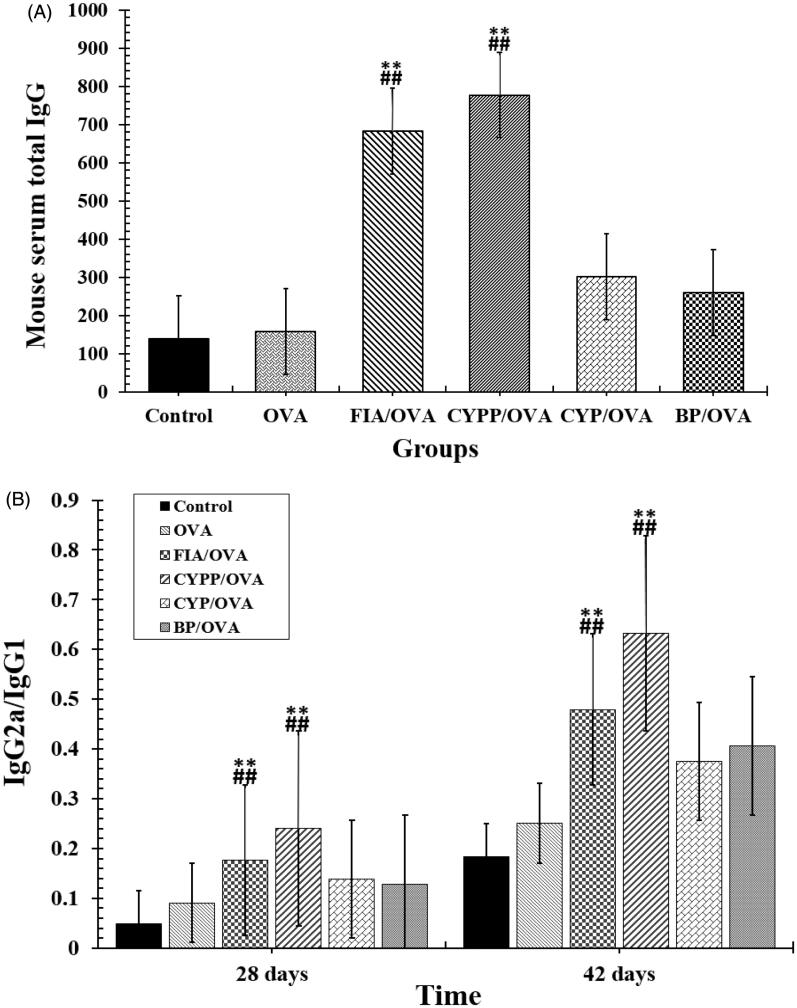
Mouse serum total IgG (A) and the ratios of IgG2a to IgG1 (B). Data are shown as the mean ± SEM. **p < .01 vs. the BP/OVA group, while ##*p* < .01 vs. the CYP/OVA group.

The effect of the subcutaneous injection of the OVA model antigen formulations on serum antibody response levels was evaluated. The serum was harvested over time from mice vaccinated using CYP mixed with OVA (CYP/OVA), OVA encapsulated within PLGA nanoparticles (BP/OVA), and CYP and OVA encapsulated within PLGA nanoparticles (CYPP/OVA). The OVA-specific IgG antibody, the mouse serum total IgG antibody, and IgG1 and IgG2a antibody titer were quantitatively evaluated using an ELISA. As depicted in Figure 4 and Figure 5(A), the CYPP/OVA induced a dramatically higher IgG antibody response on day 21 and obviously higher OVA-specific IgG antibody titers than the other formulations on day 14, 21, 28, 35, and 42 after primary immunization, which indicated that CYPP/OVA could stimulate more effective antigen-specific humoral immune responses.

The secretion of the IgG2a antibody indicates a Th1-polarized immune response, and the ratio of IgG2a/IgG1 is indicative of the level of a Th1-biased immune response (Jusforgues-Saklani et al., 2008). In Figure 5(B), both CYPP and FIA encapsulation of OVA vaccine formulations induced obviously higher IgG2a/IgG1 ratios than soluble OVA mixed with blank PLGA nanoparticles and soluble OVA mixed with CYP (*p* < .05). In addition, soluble OVA mixed with blank PLGA nanoparticles and soluble OVA mixed with CYP induced higher ratios of IgG2a/IgG1 than those induced by saline or soluble OVA alone. The result revealed that CYPP/OVA stimulated a stronger Th1-biased immune response in comparison with soluble OVA mixed with CYP alone.

### 3.5 Splenocyte proliferation assay

**Figure 6.**
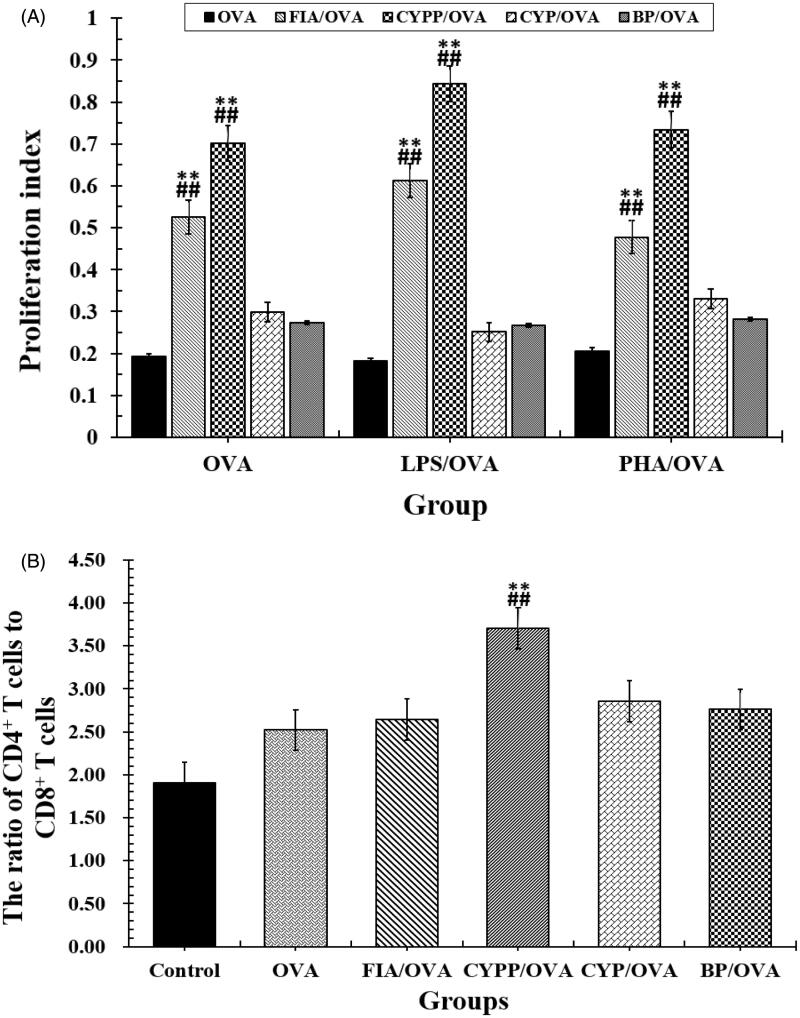
(A) Proliferation index and (B) the ratio of CD3^+^CD4^+^ cells to CD3^+^CD8^+^ cells in splenocytes harvested from vaccinated mice and re-stimulated using OVA. Data are expressed as the mean ± SEM. **p < .01 vs. the BP/OVA group, while ##*p* < .01 vs. the CYP/OVA group.

A splenocyte proliferation assay was performed to evaluate the impact of the various vaccine formulations on splenocyte proliferative responses and determine antigen-specific splenocyte activation (Liu et al., 2016). As presented in Figure 6(A), under the stimulation of OVA, the splenocytes harvested from mice immunized using the CYPP/OVA vaccine formulation proliferated more than others collected from mice immunized using soluble OVA mixed with BPs or CYP, and in particular, compared with those from mice treated using the soluble OVA alone (*p* < .05). Therefore, the CYPP/OVA vaccine formulation induced more effective antigen-specific immune responses than the other formulations.

### 3.6 The activation of T cells

Helper T (Th) cells play a major role in helping B cells to activate and produce antibodies with high avidity. The CD4 receptor is the co-receptor of the TCR and is expressed mainly on Th cells, while the CD8 receptor is expressed on cytotoxic T lymphocytes (Luo et al., 2016). The ultimate aim of vaccination is to enhance the quantity of Th cells and improve the immune effect to achieve immune protection and prevention. To compare the impact of the CYPP/OVA and CYP/OVA formulations in inducing cellular immune responses, the level of Th cells was evaluated using flow cytometry. As seen in Figure 6, a significantly higher ratio of CD4^+^ T cells to CD8^+^ T cells (all the cells were CD3^+^) was measured in the spleen of mice immunized using CYPP/OVA, compared with all of the other formulation groups. However, the other groups demonstrated negligible variations of the ratio of CD4^+^ to CD8^+^ T cells in comparison with the OVA alone group, but the ratio of CD4^+^ to CD8^+^ T cells in all test groups was significantly higher than that in the saline group. As shown in Figure 6(B), CYPP/OVA could generate a stronger cellular immune response compared with the CYP/OVA and BP/OVA, which demonstrated that the CYPP-adjuvant OVA vaccine formulation induced a Th1-biased antibody response in mice compared with that induced by soluble OVA alone.

### 3.7 Cytokine levels secreted by ex vivo re-stimulated splenocytes

**Figure 7.**
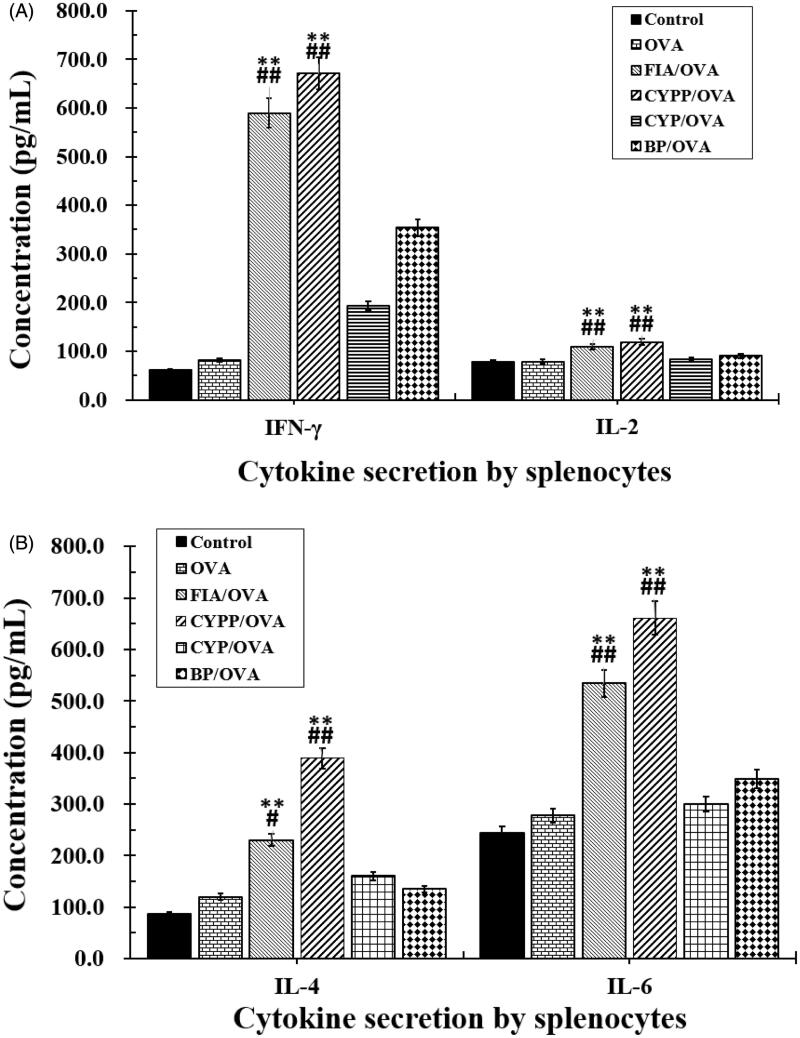
Cytokine secretion. Mice (*n* = 4) were immunized using different vaccine formulations. Splenocytes were harvested 21 d after the first immunization. For the ELISA assay, the splenocytes were re-stimulated using OVA (50 μg/mL) *in vitro*. The levels of IFN-γ and IL-2 (A), and IL-4 and IL-6 (B). Data are expressed as the mean ± SEM. *p < .05 and **p < .01 vs. the BP/OVA group, while #*p* < .05 and ##*p* < .01 vs. the CYP/OVA group.

Splenocytes collected from vaccinated mice were re-stimulated *ex vivo* using OVA, and Th1 (IFN-γ, IL-2) and Th2 (IL-4, IL-6) cytokines in the supernatant were measured using an ELISA. As shown in Figure 7(A,B), mice immunized using the CYPP/OVA formulations secreted the highest levels of IFN-γ and IL-2 compared with the other groups (*p* ≤ .05), indicating that Th1 cells were mainly activated. Moreover, the IFN-γ levels of the BP/OVA and FIA/OVA groups were obviously higher than those of the OVA alone group and the negative control group (saline group). The highest levels of IL-4 and IL-6 were secreted by splenocytes from mice vaccinated using CYPP/OVA. In general, the CYPP/OVA formulation and the FIA/OVA formulation induced higher levels of both Th1 and Th2 cytokine secretion by splenocytes, revealing stronger immune responses.

### 3.8 Expression of surface molecules on DCs in draining lymph nodes

**Figure 8.**
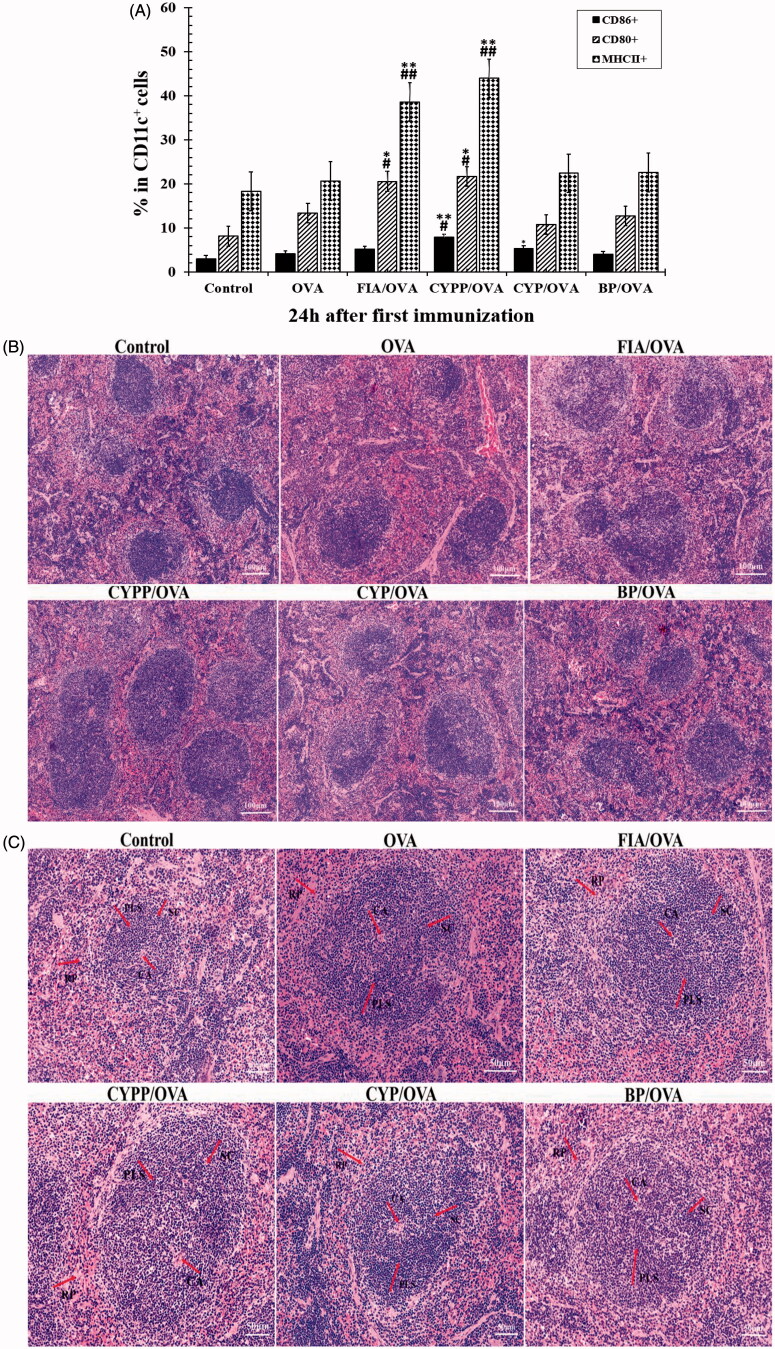
CYPP activating DCs in draining lymph nodes *in vivo*. The percentage of CD80^+^, CD86^+^, or MHC II^+^ cells (all CD11c^+^) from mice vaccinated using CYP/OVA, BP/OVA, GLP/OVA, or OVA alone, 24 h after the subcutaneous injection. Data are expressed as the mean ± SEM. *p < .05 and **p < .01 vs. the BP/OVA group, while #*p* < .05 vs. the CYP/OVA group (A). Hematoxylin–eosin staining of spleens of mice immunized using normal saline (control group), OVA alone, FIA/OVA, CYPP/OVA, BP/OVA, or CYP/OVA. CA: central artery; PLS: periarterial lymphatic sheath; SC: splenic corpuscle; RP: red pulp. Scale bar represents 100 μm (B) and 50 μm (C).

The potency of the different formulations in terms of activating DCs in the draining lymph nodes was investigated. MHC class II molecule and co-stimulatory molecule (CD80 and CD86) expression on DCs in draining lymph nodes was determined using flow cytometry. As shown in Figure 8(A), compared with soluble OVA alone, the CYPP-adjuvanted vaccine formulations induced significantly higher CD80, CD86, and MHC II expression at 24 h after first immunization; no significant differences were observed between the CYPP-adjuvanted vaccine formulations and the FIA-adjuvanted vaccine formulations. Vaccination using the CYPP-encapsulated OVA formulation resulted in a more robust response in the draining lymph nodes compared with the administration of saline or soluble OVA alone. Collectively, these data indicate that the CYPP-encapsulated OVA vaccine formulation effectively provided enough initial antigen exposure.

### 3.9 Hematoxylin–eosin staining of spleens

The spleens of the CYPP/OVA formulation-vaccinated mice demonstrated distinct changes compared with those of the other immunization groups, as displayed in Figure 8(B,C). The white pulp and red pulp boundaries of the CYPP/OVA immunized mouse spleen were distinct. Moreover, the peripheral lymphatic sheath (PLS) in the middle of the white pulp was thickened. The quantity and volume of the splenic corpuscles were increased, and the figure demonstrates that the lymphocytes and the germinal center in the CYPP/OVA-immunized mouse spleen were more obvious than in the other groups.

## 4. Discussion

A previous study reported that the CYPPs formed a homogeneous and translucent suspension. Those NPs demonstrated a similar particle size and uniform size distribution as the NPs in the current study, when observed using TEM and scanning electron microscopy (Luo et al., 2016). Several key steps are involved in the induction of protective immune responses, which include antigen uptake and processing by APCs, activation of APCs for effective T cell priming, and activation of B cells. It is well known that DCs are the most potent dedicated APCs (Banchereau et al., 2003; Banchereau & Palucka, 2005). Moreover, DCs are the major regulators of the adaptive immune response (Hartgers et al., 2000) and are the only cell type that is capable of promoting T cell proliferation. Helper T cells play an important role in helping B cells to activate and produce antibodies with high avidity (Steinman et al., 2003).

It is well known that immature DCs possess a strong endocytic capacity and express various pathogen recognition receptors, such as Toll-like receptors (TLRs), and continuously sample their surroundings for signals of danger. TLR triggering leads to phenotypical changes, facilitating Ag processing, MHC presentation, and increasing cytokine production, a process termed DC maturation (Storni & Bachmann 2004; Pasare & Medzhitov, 2005). Immature DCs transform into mature DCs that express relatively high levels of surface MHC class I and II products and co-stimulatory molecules, such as CD80 and CD86. The mature cells demonstrate a reduced capacity for antigen uptake but possess an exceptional capacity for T cell stimulation, which perhaps increase opportunities for T cell capture and interaction (Mellman & Steinman, 2001). Therefore, the level of internalization reflects the effect of the antigen presentation and the expression of surface molecules indicates the maturity and activation of the DCs, which is a prerequisite for effective antigen presentation. The NPs were prepared as adjuvants to increase the successful induction of sufficient immune responses, and the level of internalization and the variation in immunophenotype in BMDCs were determined. In addition, LPS is known as a maturational stimulus for DCs and is used as a positive control in the current research (Ardeshna et al., 2000; Park et al., 2002).

In the current study, BMDCs were used in an *in vitro* model to research the immunological function of NP-adjuvanted vaccine formulations. After the DCs were treated using free CYP, BPs, and CYPPs, the highest levels of cell internalization were observed when the DCs were cultivated with CYPPs (Figure 2). Furthermore, based on the flow cytometry data, there was a higher percentage of CD11c^+^ BMDCs that were positive for CD80, CD86, and MHC II from mice treated using the CYPPs, compared with CYP/OVA and BP/OVA, which confirms that the CYPPs demonstrate better adjuvant activity than the other formulations (Figure 3).

In the current research, the influence of various antigen vaccine formulations on antigen exposure in the immune system was evaluated and antigen-specific immune responses were assessed *in vivo*. In fact, multiple physicochemical properties of NPs inordinately affect the adjuvant effect, and one of the critical factors is the kinetics of antigen exposure to the immune system. The double emulsion solvent evaporation method and the encapsulation of antigen into PLGA NPs through ultrasonication are commonly used methods. Previous studies have demonstrated that OVA encapsulation in NPs not only protects the immunological properties of the enclosed antigen (Danhier et al., 2012), but also improves its aqueous solubility and bioavailability to cells. The efficacy of particle vaccines was reported to be significantly influenced by various physicochemical characteristics of the NPs, such as particle size and surface charge (Kohli & Alpar 2004; Foged et al., 2005). The colloidal stability of NPs/OVA was determined through measurement of particle size, PDI, zeta potential, and OVA-EE. As shown in Figure 1, the size of the CYPP/OVA at approximately 208 nm is bigger than that of the BP/OVA, at approximately 186 nm. The NPs demonstrated a low PDI (PDI <0.3), a negative surface charge, and a high OVA-EE (>90%) for the NPs/OVA, confirming that the NPs/OVA vaccination formulation possesses excellent physicochemical characteristics and is highly suited for further study.

The spleen is one of the most important immune organs. Both B and T lymphocytes are indispensable for immunologic responses. T cells mediate cellular immunity as well as being immunomodulatory. B cells primarily participate in humoral immunity (Huang et al., 2013). The proliferation of lymphocytes is the most important index reflecting organic immunity *in vivo* (Letsch & Scheibenbogen, 2003). Cytokine secretion by CD3^+^CD4^+^ Th cells plays a key role in regulating the effect of the immune response. For instance, IFN-γ and IL-2 are secreted by Th1 cells and mediate cellular immunity, while IL-4 and IL-6 are secreted by Th2 cells (Letsch & Scheibenbogen, 2003). The cellular immune response is dependent on the activation of antigen-specific CD3^+^CD4^+^ T cells and CD3^+^CD8^+^ T cells. The activated CD3^+^CD4^+^ T cells produce lots of non-overlapping sets of cytokines to mediate cytotoxic T lymphocytes and the functions of B cells (Zhu & Paul, 2008).

In the current study, the mice immunized using the CYPP/OVA vaccine formulation exhibited high avidity and enhanced induction of OVA-specific IgG and total IgG antibodies in serum (Figure 4 and Figure 5(B)), increased cytokine secretion by splenocytes (Figure 7(A,B)), and increased proliferation of splenic lymphocytes (Figure 6(A)). Moreover, the CYPP/OVA group produced the highest ratio of IgG2a/IgG1 and the highest ratio of CD3^+^CD4^+^ T cells to CD3^+^CD8^+^ T cells (Figure 5(B)), which is associated with Th1-biased immune responses, and this is attributable to the presentation of OVA encapsulated in CYPPs to APCs.

The current study confirmed that the CYPP/OVA system elicited both Th1 and Th2 immune responses for protein vaccines, with a greater Th1 bias in comparison with the FIA and the free CYP alone adjuvant formulations for protein vaccines.

The mouse spleen is composed of white pulp and red pulp. The white pulp of the spleen includes the PLS and the splenic corpuscle. The PLS is a thick layer of diffuse lymphoid tissue around the central artery and contains a large number of T cells and some macrophages in a staggered formation. T cells proliferate and the lymphatic sheath thickens in the PLS around the central artery when a cell immune response occurs. The splenic corpuscle contains a significant number of B cells, and the number of B cells is increased under antigen stimulation (Gartner & Hiatt, 2006). As shown in Figure 8(B,C), in the spleen of mice immunized using CYPP/OVA, there are obvious changes in the quantity of lymphocytes, the volume of the splenic corpuscle and the germinal center, and the thickening of the lymphatic sheath, compared with all the other groups. All of the images demonstrate that the vaccine formulation composed of antigen encapsulated in CYPPs caused the most powerful immune responses compared with the other formulations.

The ability of DCs to internalize NPs *in vitro* has been discussed in a previous section. Previous research has shown that DCs have an effective phagocytic activity that can inform the adaptive immune system, through their unique ability to sample tissue antigens, migrate to the draining lymph nodes, present extracellular antigens, and elicit tissue-specific T cell immunity (Wykes et al., 1998; Randolph et al., 2005). Therefore, different vaccine formulations were subcutaneously administered to mice and then the upregulation of surface markers in DCs in the draining lymph nodes was examined (Zhang et al., 2014; Gao et al., 2015). As shown in Figure 8(A), a higher expression of MHC II, CD80 and CD86 was observed in DCs in the draining lymph nodes at 24 h after immunization using the CYPP/OVA vaccine formulation. The results show that the activation of DCs in the drainage lymph nodes can effectively activate initial immunity and improve the effective protection of the mice, which demonstrates that immunopotentiation in mice is impacted by formulation-dependent differences (Zhang et al. 2014).

## 5. Conclusions

In a previous study, the appropriate dose of NPs was determined, which established the foundation for designing these *in vitro* and *in vivo* NP experiments. The current study investigated the impact of three different formulations on DCs *in vitro*. Both the results of the CLSM and TEM indicate that the CYPPs are more effectively internalized by DCs than free CYP and BPs. Moreover, the results of the flow cytometry indicate that immature DCs are converted to mature DCs to a greater extent through treatment using CYPPs, compared with treatment using the other formulations, indicating an enhanced ability for antigen presentation. To further explore these formulations, the current study determined whether antigen-specific immune responses are activated by the various antigen-NP formulations *in vivo*. The enhanced immune responses stimulated by the CYPP/OVA vaccine formulations might be attributed to the efficient induction of DC activation in the draining lymph nodes, enhanced induction of serum antibody titers, increased cytokine secretion by splenocytes, and the proliferation of splenic lymphocytes.

The data revealed that the vaccine formulation composed of OVA encapsulated in CYPPs stimulated the strongest antigen-specific immune responses compared with the other tested vaccine formulations. Based on these *in vitro* and *in vivo* results, the CYPPs demonstrate a strong immunoenhancement activity, which capitalizes on the impact of antigen-NP formulations on resultant immune responses. This system has significant potential and provides a theoretical basis for rational vaccine design.
